# QCD corrections to $H \rightarrow \textit{gg}$ in FDR

**DOI:** 10.1140/epjc/s10052-013-2686-1

**Published:** 2014-01-23

**Authors:** Roberto Pittau

**Affiliations:** Departamento de Física Teórica y del Cosmos and CAFPE, Universidad de Granada, Campus Fuentenueva s. n., 18071 Granada, Spain

## Abstract

I apply FDR—a recently introduced four-dimensional approach to quantum field theories (QFTs)—to the computation of the NLO QCD corrections to $H \rightarrow gg$ in the large top mass limit. The calculation involves all key ingredients of QCD—namely ultraviolet, infrared, and collinear divergences, besides $\alpha _S$ renormalization—and paves the way for successful use of FDR in massless one-loop QFT computations. I show in detail how the correct result emerges in FDR, and discuss the translation rules to dimensional regularization.

## Introduction

Many of the difficulties of higher-order calculations in QFT can be traced back to the treatment, in the framework of dimensional regularization (DR) [1], of the infinities arising in the intermediate steps of the computation. Ultraviolet (UV), infrared (IR), and collinear (CL) divergences are first dimensionally regulated, and then renormalized away—in the UV case—or canceled by combining virtual and real contributions, or reabsorbed in the collinear behavior of the initial state parton densities. In order to attack the problem numerically, it is often necessary to subtract and add back approximations of the IR/CL singular structures. At one loop, several well-tested subtraction procedures have been introduced in the last two decades [2–8]. At two loops and beyond, the situation is more involved, but progress is under way [9–14].

The first obvious ingredient, which may lead to a significant simplification in the above picture, is a computational procedure in which all parts of the calculation can be directly treated in four dimensions. As for the virtual contribution, the FDR approach has been recently introduced in reference [15], which allows a subtraction of the UV divergencesat the level of the integrand, leaving a four-dimensional integration over the loop momenta. In the same work, the use of FDR as an IR regulator in QED with massive fermions is also suggested.

In this paper, I present the first application of the FDR ideas in the context of fully massless QCD, where the issues related to gauge invariance are much more subtle than in the QED case. I concentrate, in particular, on the calculation of the $\mathcal{O}(\alpha _S)$ gluonic corrections to the $H \rightarrow gg$ decay in the $m_\mathrm{top} \rightarrow \infty$ limit, and re-derive the well-known fully inclusive result [16, 17]

1 $$\begin{aligned} \varGamma (H \rightarrow gg) = \varGamma ^{(0)}(\alpha _S(M_H^2)) \left[ 1+\frac{95}{4}\,\frac{\alpha _S}{\pi } \right] , \end{aligned}$$

where

2 $$\begin{aligned} \varGamma ^{(0)}(\alpha _S(M_H^2))= \frac{G_F \alpha _S^2(M_H^2)}{36 \sqrt{2} \pi ^3} M^3_H \end{aligned}$$

is the lowest order contribution, with $N_F= 0$ in $\alpha _S(M_H^2)$, since only gluons are considered.

Despite its simplicity, all key ingredients of massless QCD are present in this process, such as the simultaneous occurrence of IR/CL divergences and UV renormalization. The fact that the correct expression is reproduced shows that FDR is a valid and consistent approach in massless QFTs, and it gives confidence in its potential to simplify multi-leg/loop computations.

The outline of the paper is as follows. Section 2 provides the set-up of the calculation. In Sect. 3, I review the FDR treatment of the UV divergences and discuss its interplay with the IR and CL infinities. Section 4 presents the FDR computation of the virtual part, while Sect. 5 deals with the real contribution and its merging with the one-loop piece. The connection between FDR and DR is discussed in Sect. 6 and the final conclusions are drawn in Sect. 7.

## The model for $H \rightarrow gg$

**Fig. 1:**
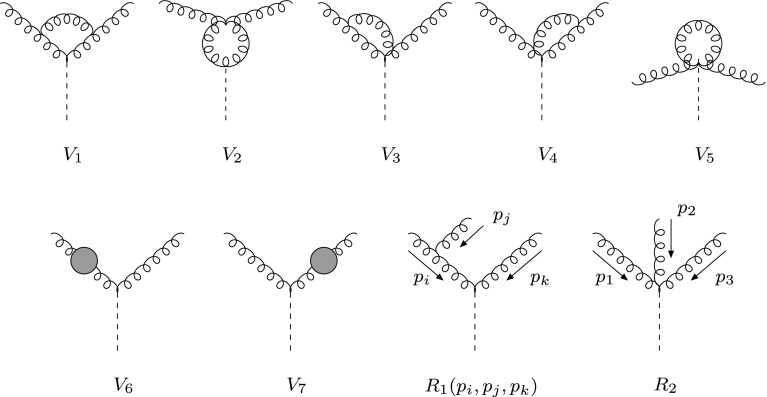
Virtual and real diagrams contributing to $H \rightarrow g g (g)$ at $\mathcal{O}(\alpha _S^3)$. The *gray blobs* in $V_6$ and $V_7$ represent gluon wave-function corrections and the *dashed line* stands for the Higgs field. $R_1(p_i,p_j,p_k)$ corresponds to three diagrams with permuted gluons

The effective interaction of one Higgs field $H$ with two, three and four gluons—mediated by an infinitely heavy top loop—is described by the Lagrangian [18, 19]

3 $$\begin{aligned} \mathcal{L}_\mathrm{eff} = -\frac{1}{4} A H G^{a}_{\mu \nu } G^{a,\mu \nu }, \end{aligned}$$

where

4 $$\begin{aligned} A = \frac{\alpha _S}{3\pi v}\left( 1+\frac{11}{4}\frac{\alpha _S}{\pi } \right) \end{aligned}$$

and $v$ is the vacuum expectation value, $v^2= (G_F \sqrt{2})^{-1}$. The corresponding Feynman rules are given in [20], and the diagrams for the decay rate $\varGamma (H \rightarrow gg)$ are drawn in Fig. 1.

There are five graphs contributing to the virtual part $\varGamma _{V}$—without counting gluon wave-function corrections—and four diagrams for the real radiation $\varGamma _{R}$. In the following, I separately compute, in FDR, the two pieces, showing how IR/CL divergences drop in the sum

5 $$\begin{aligned} \varGamma _{V}(H \rightarrow gg) + \varGamma _{R}(H \rightarrow ggg). \end{aligned}$$

## FDR versus infinities

The FDR subtraction of UV infinities is better illustrated with an explicit example. Consider the one-loop quadratically divergent rank-two tensor

6 $$\begin{aligned} \int d^4q \frac{q_\alpha q_\beta }{D_0 D_1}, \end{aligned}$$

with

7 $$\begin{aligned} D_i ~=~ q^2-d_i,~~~d_i ~=~ M^2_i-p^2_i- 2 (q\cdot p_i),~~~p_0=0.\nonumber \\ \end{aligned}$$

Its UV convergence can be improved by first deforming the propagators by a vanishing amount $\mu ^2$ 1

8 $$\begin{aligned} D_i \rightarrow \bar{D}_i~=~D_i-\mu ^2, \end{aligned}$$

and then by repeatedly using the identity

9 $$\begin{aligned} \frac{1}{\bar{D}_i} = \frac{1}{\bar{q}^2} \Bigg (1+\frac{d_i}{\bar{D}_i} \Bigg ), \end{aligned}$$

where

10 $$\begin{aligned} \bar{q}^2~=~q^2-\mu ^2. \end{aligned}$$

Note that the prescription in Eq. (8) avoids possible infrared divergences in the r.h.s. of Eq. (9). The integrand in Eq. (6) can then be rewritten as

11 $$\begin{aligned} \frac{q_\alpha q_\beta }{\bar{D}_0 \bar{D}_1}&= q_\alpha q_\beta \left( \left[ \frac{1}{\bar{q}^4} \right] +\left[ \frac{d_0+d_1}{\bar{q}^6} \right] +\left[ \frac{d_1^2}{\bar{q}^8} \right] +\frac{d_1^3}{\bar{q}^8 \bar{D}_1} \right. \nonumber \\&\left. + \frac{d_0 d_1}{\bar{q}^6 \bar{D}_1} + \frac{d_0^2}{\bar{q}^4 \bar{D}_0\bar{D}_1} \right) , \end{aligned}$$

where the terms in square brackets are UV divergent but depend only on $\mu ^2$. The FDR *definition* of the integral in Eq. (6) is obtained by integrating the expansion in Eq. (11), after dropping the divergent pieces, and taking the physical limit $\mu \rightarrow 0$:

12 $$\begin{aligned}&B_{\alpha \beta }(p_1^2,M_0^2,M_1^2) = \int [d^4q] \frac{q_\alpha q_\beta }{\bar{D}_0 \bar{D}_1} \nonumber \\&\quad \equiv \lim _{\mu \rightarrow 0} \int d^4q \, q_\alpha q_\beta \left( \frac{d_1^3}{\bar{q}^8 \bar{D}_1} + \frac{d_0 d_1}{\bar{q}^6 \bar{D}_1} + \frac{d_0^2}{\bar{q}^4 \bar{D}_0\bar{D}_1} \right) \!.\nonumber \\ \end{aligned}$$

The r.h.s. of Eq. (12) corresponds to a well-defined four-dimensional integral, in which all UV divergences are explicitly subtracted. Furthermore, IR and CL divergences get also regulated by the propagator deformation. The gauge-invariance properties of this definition are discussed in detail in [15, 21]. In the rest of this section, I mostly concentrate on CL and IR infinities, and, in particular, on the matching between virtual and real contributions.

A convenient starting point to study the CL singularities is the fully massless limit of Eq. (12):

13 $$\begin{aligned}&B_{\alpha \beta }(0,0,0) = \lim _{\mu \rightarrow 0} \int d^4q \, \frac{q_\alpha q_\beta d_1^3}{\bar{q}^8 \bar{D}_1} \nonumber \\&\quad = -8 p_1^\rho p_1^\sigma p_1^\tau \lim _{\mu \rightarrow 0} \int d^4q \frac{q_\alpha q_\beta q_\rho q_\sigma q_\tau }{\bar{q}^8 \bar{D}_1} = 0, \end{aligned}$$

which vanishes, after tensor decomposition, since $p_1^2= 0$. Analogously, one proves that

14 $$\begin{aligned}&B_{\alpha }(0,0,0) = \int [d^4q] \frac{q_\alpha }{\bar{D}_0 \bar{D}_1} = 0, \nonumber \\&B(0,0,0) = \int [d^4q] \frac{1}{\bar{D}_0 \bar{D}_1} = 0. \end{aligned}$$

Those results coincide with DR—in which scale-less integrals are zero [22]—and are due to a cancelation between two $\ln (\mu ^2)$ of CL and UV origin, respectively. For example,

15 $$\begin{aligned} B(p^2,0,0)&= -i \pi ^2 \lim _{\mu \rightarrow 0}\int _0^1 \mathrm{d}x\, \nonumber \\&\times \,\,\left[ \ln (\mu ^2 -p^2 x (1-x)) -\ln (\mu ^2) \right] \!, \end{aligned}$$

where the first logarithm develops a CL singularity in the limit $p^2 \rightarrow 0$.

**Fig. 2:**
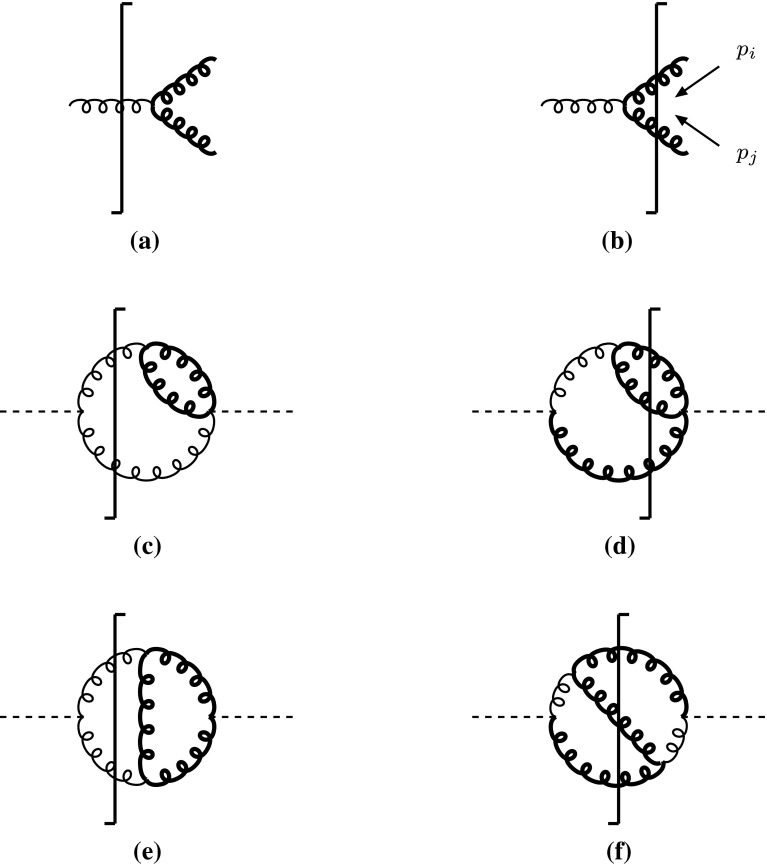
Gluon splitting IR/CL singularities regulated by massive (thick) gluons. The one-gluon cut in **a** contributes to the virtual part, the two-gluon cut in **b** to the real radiation. **c**–**f** represent typical cut-diagrams contributing to $H \rightarrow gg(g)$

Thus, the virtual CL infinities, generated by $1 \rightarrow 2$ splittings of massless particles, are naturally regulated by the $\mu ^2$-deformed propagators inside the loop, while the external momenta remain massless, as illustrated in Fig. 2 a and c. The real counterpart of this procedure is exemplified in Fig. 2b and d, and corresponds to a phase space in which all would be massless external particles are given a common mass $\mu$ and the internal ones stay massless. In other words, one has to replace^2^

16 $$\begin{aligned} \frac{1}{2(p_i \cdot p_j)} \rightarrow \frac{1}{(p_i+p_j)^2} \end{aligned}$$

in any possible singular denominator of the real matrix element squared, integrate over the aforementioned massive phase space, and take the limit $\mu \rightarrow 0$.

As for the IR divergences, the reasoning follows the same lines. For example, the only IR/CL divergent scalar one-loop three-point function generated by the cut in Fig. 2e is^3^

17 $$\begin{aligned} C(s)= \int [d^4q] \frac{1}{\bar{D}_0 \bar{D}_1 \bar{D}_2} = \lim _{\mu \rightarrow 0} \int d^4q \frac{1}{\bar{D}_0 \bar{D}_1 \bar{D}_2}, \end{aligned}$$

with

18 $$\begin{aligned} M_0^2 = M_1^2 = M_2^2 = p_1^2= p_2^2= 0,~~~s= -2(p_1 \cdot p_2).\nonumber \\ \end{aligned}$$

By denoting

19 $$\begin{aligned} \mu _0=\frac{\mu ^2}{s}, \end{aligned}$$

one computes

20 $$\begin{aligned} C(s)&= \lim _{\mu \rightarrow 0} \frac{i \pi ^2}{2 s} \ln ^2\Bigg (\frac{\sqrt{1-4\mu _0}+1}{\sqrt{1-4\mu _0}-1}\Bigg ) \nonumber \\&= \frac{i \pi ^2}{s} \left[ \frac{\ln ^2(\mu _0)-\pi ^2}{2} + i\,\pi \ln (\mu _0) \right] , \end{aligned}$$

which is indeed fully matched by the inclusive real contribution in Fig. 2f, as will be shown in Sect. 6.

In the following, I use the described approach to UV/CL/IR infinities to compute $\varGamma _{V}(H \rightarrow gg)$ and $\varGamma _{R}(H \rightarrow ggg)$.

## The virtual part $\varGamma _{V}(H \rightarrow gg)$

The calculation is greatly simplified by Eqs. (13) and (14). In fact, only the diagrams $V_1$ and $V_2$ in Fig. 1 contribute—as in DR—and the gluon wave function corrections vanish. One computes

21 $$\begin{aligned} \varGamma _{V}(H \rightarrow gg)=-3 \frac{\alpha _S}{\pi }\,\varGamma ^{(0)}(\alpha _S)\,M^2_H\, \mathcal{R}e \left[ \frac{C(M^2_H)}{i \pi ^2}\right] .\nonumber \\ \end{aligned}$$

This simple expression is obtained after a standard Passarino–Veltman [23] decomposition, the only subtlety being the FDR treatment of $\mu ^2$ [15, 21]: for consistency with Eq. (8), a $q^2$ appearing in the numerator of a diagram should also be deformed,

22 $$\begin{aligned} q^2 \rightarrow \bar{q}^2, \end{aligned}$$

and integrals involving $\mu ^2$, such as

23 $$\begin{aligned} {\tilde{B}}(p_1^2,M_0^2,M_1^2) = \int [d^4q] \frac{\mu ^2}{\bar{D}_0 \bar{D}_1}, \end{aligned}$$

require the same integrand expansion *as if* $\mu ^2= q^2$. For example, from Eq. (12),

24 $$\begin{aligned}&{\tilde{B}}(p_1^2,M_0^2,M_1^2)\nonumber \\&\quad = \lim _{\mu \rightarrow 0} \int d^4q \, \mu ^2 \left( \frac{d_1^3}{\bar{q}^8 \bar{D}_1} + \frac{d_0 d_1}{\bar{q}^6 \bar{D}_1} + \frac{d_0^2}{\bar{q}^4 \bar{D}_0\bar{D}_1} \right) \nonumber \\&\quad = \frac{i \pi ^2}{2}\left( M_0^2+M_1^2-\frac{p_1^2}{3}\right) . \end{aligned}$$

The final result follows by inserting Eq. (20) into (21),

25 $$\begin{aligned} \varGamma _{V}(H \rightarrow gg)=\frac{3}{2} \frac{\alpha _S}{\pi }\,\varGamma ^{(0)}(\alpha _S)\, \left( \pi ^2-\ln ^2\frac{M^2_H}{\mu ^2} \right) . \end{aligned}$$

## The real radiation $\varGamma _{R}(H \rightarrow ggg)$ and the fully inclusive result

The unpolarized matrix element squared, derived from the real emission diagrams in Fig. 1, reads

26 $$\begin{aligned}&|M|^2 = 192\,\pi \alpha _SA^2 \Bigg [\frac{s^3_{23}}{s_{12}s_{13}} +\frac{s^3_{13}}{s_{12}s_{23}} +\frac{s^3_{12}}{s_{13}s_{23}} \nonumber \\&\quad +\,\, \frac{2(s^2_{13}+s^2_{23})+3 s_{13}s_{23}}{s_{12}} + \frac{2(s^2_{12}+s^2_{23})+3 s_{12}s_{23}}{s_{13}} \nonumber \\&\quad +\,\, \frac{2(s^2_{12}+s^2_{13})+3 s_{12}s_{13}}{s_{23}}+ 6 (s_{12} + s_{13} + s_{23})\Bigg ], \end{aligned}$$

where $s_{ij}= (p_i+ p_j)^2$. This expression is obtained from the massless result with the replacement $2(p_i \cdot p_j) \rightarrow s_{ij}$, in accordance with Eq. (16). As described in Sect. 3, in order to match the virtual IR/CL singularities, $|M|^2$ should be integrated over a massive three-gluon phase space with $p_i^2= \mu ^2$, which can be parametrized as

27 $$\begin{aligned} \int d \Phi _3 = \frac{\pi ^2}{4s} \int ds_{12} ds_{13} ds_{23}\, \delta (s-s_{12}-s_{13}-s_{23}+3\mu ^2), \end{aligned}$$

where $\sqrt{s}$ is the Higgs mass. It is convenient to introduce the dimensionless variables

28 $$\begin{aligned} x= \frac{s_{12}}{s}-\mu _0,~~y= \frac{s_{13}}{s}-\mu _0,~~z= \frac{s_{23}}{s}-\mu _0, \end{aligned}$$

with $\mu _0$ given in Eq. (19), in terms of which, using the condition,

29 $$\begin{aligned} x+y+z = 1, \end{aligned}$$

all IR/CL divergent bremsstrahlung integrals can be reduced to the following ones:

30 $$\begin{aligned}&I(s) = \int _{R} \mathrm{d}x \mathrm{d}y\, \frac{1}{(x+\mu _0) (y+\mu _0)}, \nonumber \\&J_{p}(s) = \int _{R} \mathrm{d}x \mathrm{d}y\, \frac{x^p}{(y+\mu _0)}\,\,~~(p \ge 0), \end{aligned}$$

where the integration region reads, in the fully inclusive case,

31 $$\begin{aligned} \int _{R} \mathrm{d}x \mathrm{d}y \equiv \int _{3 \mu _0}^{1-2\sqrt{\mu _0}} \mathrm{d}x \int _{y_-}^{y_+} \mathrm{d}y, \end{aligned}$$

with

32 $$\begin{aligned} y_{\pm }&= \frac{1}{4(x + \mu _0)} \left[ (1-\mu _0)^2-(R_0 \mp R_1)^2\right] -\mu _0, \nonumber \\ R_0&= \sqrt{(x-\mu _0)^2-4 \mu _0^2},~~~R_1 ~=~ \sqrt{(1-x)^2-4 \mu _0}.\nonumber \\ \end{aligned}$$

Thus

33 $$\begin{aligned}&\varGamma _{R}(H \rightarrow ggg)= 3 \frac{\alpha _S}{\pi }\,\varGamma ^{(0)}(\alpha _S)\, \nonumber \\&\quad \times \left( \frac{1}{4}+I(M^2_H)-\frac{3}{2}J_0(M^2_H)-J_2(M^2_H) \right) . \end{aligned}$$

Finally, one computes, up to terms which vanish in the limit $\mu _0 \rightarrow 0$,

34 $$\begin{aligned} I(s) = \frac{\ln ^2(\mu _0)-\pi ^2}{2} \end{aligned}$$

and

35 $$\begin{aligned}&J_{p}(s) = -\frac{1}{p+1} \ln (\mu _0) + \int _0^1 \mathrm{d}x \,x^p\left[ \ln (x)+ 2 \ln (1-x) \right] \nonumber \\&\quad = -\frac{1}{p+1} \ln (\mu _0) \!-\!\frac{1}{p+1}\left[ \frac{1}{p+1}\!+\!2 \sum _{n=1}^{p+1}\frac{1}{n} \right] (p \ge 0),\nonumber \\ \end{aligned}$$

so that

36 $$\begin{aligned}&\varGamma _{R}(H \rightarrow ggg)= \frac{3}{2} \frac{\alpha _S}{\pi }\,\varGamma ^{(0)}(\alpha _S)\, \nonumber \\&\quad \times \left( \ln ^2\frac{M^2_H}{\mu ^2}-\pi ^2+\frac{73}{6} -\frac{11}{3}\ln \frac{M^2_H}{\mu ^2} \right) . \end{aligned}$$

Adding this to Eq. (25), and accounting for the finite renormalization term in Eq. (4), one obtains

37 $$\begin{aligned} \varGamma (H \rightarrow gg) = \varGamma ^{(0)}(\alpha _S) \left[ 1+\frac{\alpha _S}{\pi } \left( \frac{95}{4}-\frac{11}{2} \ln \frac{M^2_H}{\mu ^2} \right) \right] .\nonumber \\ \end{aligned}$$

All CL/IR $\ln (\mu ^2)$ and $\ln ^2(\mu ^2)$ cancel in Eq. (37), so that the remaining $\mu$ is directly interpreted as the renormalization scale. This is a typical procedure in FDR: since the UV infinities are subtracted from the very beginning, the unphysical left-over $\mu$ dependence is eliminated, on the perturbative level one is working at, by a finite renormalization, which fixes the bare parameters in terms of the observables [24]. This is obtained, in the case at hand, by simply replacing $\varGamma ^{(0)}(\alpha _S) \rightarrow \varGamma ^{(0)}(\alpha _S(\mu ^2))$ 4 in Eq. (37). Then the logarithm is reabsorbed in the gluonic running of the strong coupling constant,

38 $$\begin{aligned} \alpha _S(M_H^2)= \frac{\alpha _S(\mu ^2)}{1+\frac{\alpha _S}{2 \pi }\frac{11}{2} \ln \frac{M_H^2}{\mu ^2}}, \end{aligned}$$

and Eq. (1) follows.

## FDR versus DR

In this section, I discuss the transition rules between FDR and DR. This is particularly important in QCD, where NLO calculations have to be matched with the running of $\alpha _S$ and parton densities, conventionally derived in DR. I consider UV, CL, and IR divergences in turn, showing the equivalence of FDR with the dimensional reduction [25] version of DR, widely used in supersymmetric theories.

I start by establishing the connection between the $1/\epsilon$ DR regulator and the $\ln (\mu ^2)$ appearing in FDR. As for the UV infinities, it is sufficient to compare the FDR and DR variants of any divergent integral. For instance, the DR counterpart of Eq. (15) (with $p^2 \ne 0$) reads

39 $$\begin{aligned} \int d^nq \frac{1}{q^2(q+p)^2}= i \pi ^2 \int _{0}^{1} \mathrm{d}x\, [\Delta -\ln (-p^2 x (1-x))],\nonumber \\ \end{aligned}$$

where

40 $$\begin{aligned} n = 4 + \epsilon \,~~~\mathrm{and}~~~\Delta = -\frac{2}{\epsilon } - \gamma _E -\ln \pi . \end{aligned}$$

Thus, DR and FDR UV regulators are linked through the simple $\overline{\mathrm{MS}}$ replacement

41 $$\begin{aligned} \Delta \rightarrow \ln (\mu ^2). \end{aligned}$$

CL virtual singularities follow the same pattern, as can be inferred from the exact UV/CL cancelation in Eqs. (13) and (14). As a consistency check, the DR version of $J_{p}$ reads

42 $$\begin{aligned}&J^\mathrm{DR}_{p}(s) \!=\! \frac{(\pi s)^{\frac{\epsilon }{2}}}{\varGamma \left( 1+\frac{\epsilon }{2}\right) } \int \mathrm{d}x\,\mathrm{d}y\,\mathrm{d}z\,\frac{x^p}{y}\delta (1\!-\!x\!-\!y\!-\!z) (xyz)^{\frac{\epsilon }{2}} \nonumber \\&\quad = -\frac{1}{p+1} (\Delta -\ln (s)) -\frac{1}{p+1}\left[ \frac{1}{p+1}+2 \sum _{n=1}^{p+1}\frac{1}{n} \right] ,\nonumber \\ \end{aligned}$$

which indeed coincides with Eq. (35) if $\Delta = \ln (\mu ^2)$.

Finally, the $\ln ^2(\mu ^2)$ terms—generated by overlapping IR/CL singularities—drop, together with the full constant part, when adding virtual and fully inclusive real contributions, which can be traced back to the following relation:

43 $$\begin{aligned} \mathcal{R}e \left[ \frac{C(s)}{i \pi ^2}\right] = \frac{1}{s} I(s)\, \end{aligned}$$

between Eqs. (20) and (34). An easy calculation shows that the same happens in DR. In fact

44 $$\begin{aligned}&\mathcal{R}e \left[ \frac{1}{i \pi ^2} \int d^nq \frac{1}{q^2(q+p_1)^2(q+p_2)^2} \right] \nonumber \\&\quad = \frac{1}{s} (\pi s)^{\frac{\epsilon }{2}}\, \varGamma \left( 1-\frac{\epsilon }{2}\right) \left[ \frac{4}{\epsilon ^2}-\frac{2}{3} \pi ^2 \right] , \end{aligned}$$

where $p_1^2= p_2^2= 0$ and $s= -2(p_1\cdot p_2)$, and

45 $$\begin{aligned} I^\mathrm{DR}(s)&= \frac{(\pi s)^{\frac{\epsilon }{2}}}{\varGamma \left( 1+\frac{\epsilon }{2}\right) } \int \mathrm{d}x\,\mathrm{d}y\,\mathrm{d}z\,\frac{1}{xy}\delta (1\!-\!x\!-\!y\!-\!z) (xyz)^{\frac{\epsilon }{2}} \nonumber \\&= (\pi s)^{\frac{\epsilon }{2}}\,\varGamma \left( 1-\frac{\epsilon }{2}\right) \left[ \frac{4}{\epsilon ^2}-\frac{2}{3} \pi ^2 \right] . \end{aligned}$$

In summary, Eq. (41) is the only relation needed between the two regulators. However, an important difference between DR and FDR follows from self-contractions of metric tensors coming from the Feynman rules. In DR $g_{\alpha \beta }g^{\alpha \beta }= n$, while $g_{\alpha \beta }g^{\alpha \beta }= 4$ in FDR. This, together with Eq. (41), and the FDR treatment of $\mu ^2$ discussed in Sect. 4, makes explicit the equivalence between FDR and dimensional reduction in the $\overline{\mathrm{\mathrm MS}}$ scheme. Having established this, all the well-known transition rules between dimensional reduction and DR [26, 27] can be directly applied to FDR. In the case of Eq. (37), it turns out that the expression is the same in both dimensional reduction (or FDR) and DR. Therefore, the correct strong coupling constant to be used is the customary $\alpha _S(\mu ^2)$ in the $\overline{\mathrm{MS}}$ scheme, proving that the FDR result coincides with Eq. (1).

## Conclusions

I have presented an FDR calculation of the gluonic QCD corrections to $H \rightarrow gg$ in the large top effective theory, demonstrating that ultraviolet, collinear, and infrared divergences can be simultaneously and successfully regulated in four dimensions. I have proved the equivalence, at the one-loop level, of dimensional reduction and FDR, making the latter approach attractive also in supersymmetric calculations, where the fermionic and bosonic sectors must share the same number of degrees of freedom.

The advantage of directly working in the four-dimensional Minkowsky space is expected to lead to considerable simplifications in higher-order QFT computations, especially in connection with numerical techniques. This issue, together with the extension of FDR to more loops, is currently under study.

## Footnotes

1
$-\mu ^2$ can be directly identified with the $+ i 0$ propagator prescription.

2 See Fig. 2b.

3 The FDR integration corresponds to a normal integration, in this case, because $C(s)$ is UV finite.

4
$\alpha _S(\mu ^2)$ has to be computed in the $\overline{\mathrm{MS}}$ scheme, as explained in the next section.
